# Diagnostic Difficulties in Pathological Laboratories in Developing Countries: A Case Report of Differentiated Squamous Cell Carcinoma in a Young Togolese Woman

**DOI:** 10.1155/2016/3727484

**Published:** 2016-03-15

**Authors:** Tchin Darré, Lantam Sonhaye, Mazamaesso Tchaou, Abdoul-Samadou Aboubakari, Bingo K. M'Bortche, Kofi Amégbor, Gado Napo-Koura

**Affiliations:** ^1^Department of Pathology, The University Teaching Hospital of Lomé, Togo; ^2^Department of Imaging, The University Teaching Hospital of Lomé, Togo; ^3^Department of Obstetrics and Gynecology, The University Teaching Hospital of Lomé, Togo

## Abstract

We report a case of poorly differentiated squamous cell carcinoma of the vulva induced by human papillomavirus in a 23-year-old woman, in whom we experienced diagnostic difficulties.

## 1. Introduction

Vulva cancers are very rare and serious tumors [1]. Infection with human papillomavirus (HPV) is the main risk factor in the occurrence of vulva cancers, especially in young women [2]. We report a case of poorly differentiated squamous cell carcinoma of the vulva in a 23-year-old young woman due to HPV, genotypes 16/18 and 6/11, detected by immunohistochemistry and in situ hybridization. We encountered difficulties in the process of diagnosing this differentiated squamous cell carcinoma of the vulva.

## 2. Case Report

In January 2014, a 23-year-old unmarried woman consulted gynecology department of the “Sylvanus Olympio Teaching Hospital in Lomé” for a painful swelling of the major labia of the vulva. She had her first menstruations at 16 years of age and her first sexual intercourse at 20 years of age. She did not report any history of sexually transmitted infections. She did not smoke, nor take alcohol, nor take contraceptive pill.

Clinical examination revealed axillary temperature of 37°C, body weight of 75 Kg, and height of 1.65 m. Gynecological examination noted, at the major labia of the vulva, a firm swelling of about 2 cm, with regular contours. The major labium of the vulva was painful at contact. There were no satellite adenopathies.

Clinicians established an initial diagnosis of inflammation of Bartholin gland. A tumorectomy was done in February 2014 and the removed piece was sent for histological analysis. Conventional histological analysis of a biopsy performed in February 2014 concluded to a botryoid sarcoma of the vulva. Also, paraffin blocks were sent to France for specific examinations.

**Figure 1:**
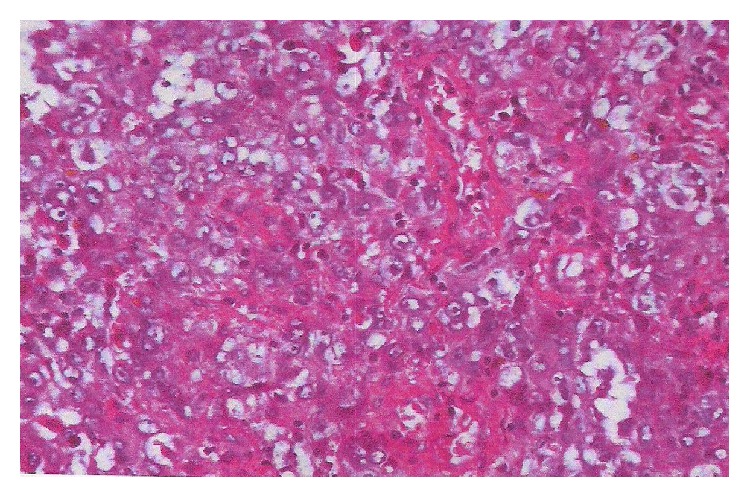
Poorly differentiated squamous cell carcinoma of the vulva (HE, ×250).

**Figure 2:**
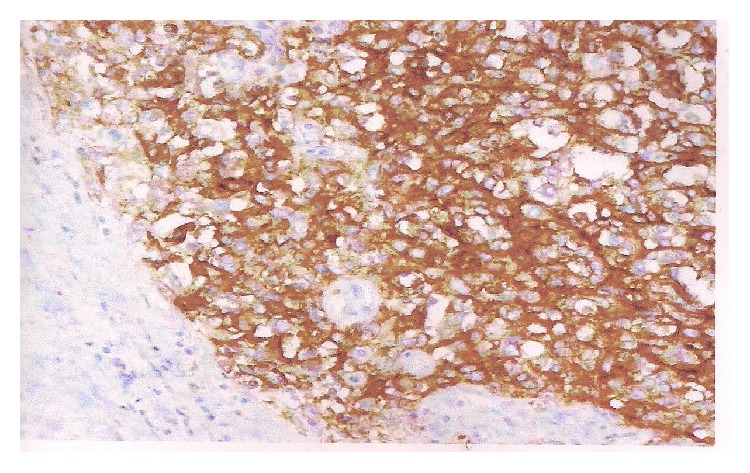
Diffuse positivity for anti-EMA tumor cells in immunohistochemistry.

**Figure 3:**
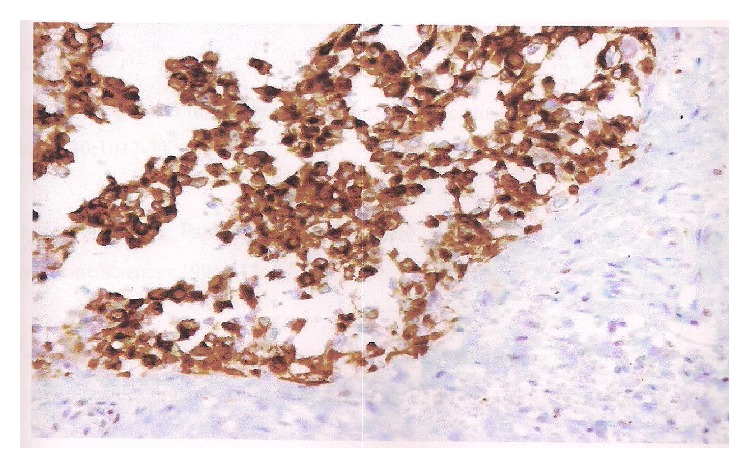
Focal positivity for KL1 type of anti-cytokeratin tumor cells in immunohistochemistry, anti-cytokeratin.

Immunohistochemistry investigations showed positivity of anti-epithelial membrane antigen (EMA) markers and anti-pancytokeratin 1 markers of tumor cells, but they were negative for anti-smooth muscle actin and desmin protein S100 ones (Figures 1, 2, and 3).

In situ hybridization revealed the HPV 16/18 and 6/11 genotypes. The diagnosis of squamous cell carcinoma with poorly differentiated sequences of HPV was established. Chemotherapy was instituted.

**Figure 4:**
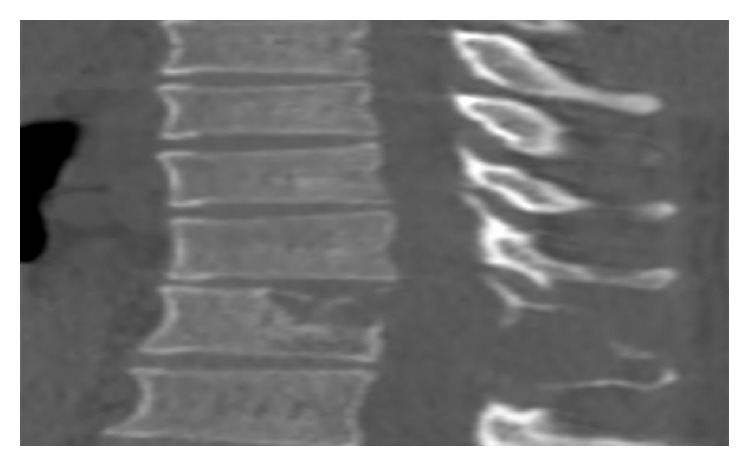
Metastatic vertebral osteolysis spondyle and the spinous process of the twelfth thoracic vertebra.

One year after the beginning of chemotherapy, the patient complained of abdominal and bone pain. Thoracolumbar spine scan showed a metastatic osteolysis of vertebral spondyle and spinous process of the twelfth thoracic vertebra (Figure 4). Abdominal ultrasound had shown no abnormalities. After multidisciplinary discussions, radiotherapy was decided in addition to the chemotherapy.

## 3. Comments

Our reported case illustrates the diagnostic difficulties encountered in practice by the pathological laboratories in sub-Saharan Africa (absence of immunodetection techniques, lack of electron microscopy, and in situ hybridization). In fact, in our case, without the collaboration with a pathological laboratory in France, this diagnosis of squamous cell carcinoma would not have been established. Many studies have confirmed the role of oncogenic HPV in the genesis of neoplasia, as with squamous privileged location [1, 3]. Infections with HPV 16 and 18 are associated with a major risk of genital cancers, causing an increase in the frequency of these cancers [4].

Our clinical cases of squamous cell carcinoma cancer concerned a young woman who showed no particular medical history and who developed a cancer 3 years after her first sexual intercourse. However, the vulvae cancer is observed in the elderly between 6th and 7th decade with mucosa estrogen deficiency, according to the literature [1, 3]. In a study, Zekan et al. found 28% of HPV in the age group of 20–22 years and the induction period of cancer was estimated between 20 and 30 years after the first sexual intercourse [5]. According to Kokka et al., a woman who had her first sexual intercourse before the age of 16 years has a risk of an oncogenic HPV infection twice higher than the one whose first intercourse occurred after the age of 20 years [6]. The patient in our study reported to have had her first sexual intercourse at 20 years old and as such was deemed to have a lower risk of developing carcinoma. The risk of developing genital cancer is three times higher in women with almost ten sexual partners than in those having one partner [3]. In our case, the woman had only one sexual partner since her first sexual intercourse.

HPV infection and genital cancers are now considered as opportunistic infections in patients with human immunodeficiency virus [5]. Histologically, cancer of the vulva is a squamous cell cancer in 90% of cases and sarcomas or melanocarcinomas in 8% and other histological types are met in 2% [1]. It is known that immunohistochemical analyses have several advantages in the diagnosis: the positivity of cytokeratin markers and of the epithelial membrane antigen reflects the epithelial nature of this cancer, and the negativity of desmin and smooth muscle actin protein S100 markers allows excluding the diagnosis of sarcoma botrytis. In situ hybridization has allowed isolating the types of HPV. Usually, it is the low potential oncogenic papillomavirus types 6, 11, 42, and 55 which are found in the anogenital region [6]. The poorly differentiated carcinoma due to HPV is consistent with the opinion of Xu et al. and others who think that the papillomavirus is the instrument of pathology of undifferentiated vulva lesions [7].

On therapeutic side, treatment of this cancer is primarily surgical. Our patient received only chemotherapy with disappointing outcomes. Radiotherapy was required in our patient because chemotherapy combined with radiation therapy is often recommended for extended forms [4].

## 4. Conclusion

This case report shows the diagnostic difficulties of infections related to papillomavirus encountered by pathology laboratories in developing countries and poses the problem of providing adequate therapeutic care of such cancers in our country.
